# miR-582-5P induces colorectal cancer cell proliferation by targeting adenomatous polyposis coli

**DOI:** 10.1186/s12957-016-0984-4

**Published:** 2016-09-06

**Authors:** Zhenbo Shu, Libo Chen, Dayong Ding

**Affiliations:** 1 Department of Gastrointestinal Colorectal and Anal Surgery, China-Japan Union Hospital, Jilin University, ChangChun, 130033 China; 2 Department of Ultrasound, China-Japan Union Hospital, Jilin University, ChangChun, 130033 China

**Keywords:** miR-582-5P, APC, Proliferation, Colorectal cancer

## Abstract

**Background:**

microRNA (miRNAs) dysregulation is widely involved in cancer progression and contributed to sustained cell proliferation by directly targeting multiple targets. Therefore, better understand the underlying mechanism of miRNA in carcinogenesis may improve diagnostic and therapeutic strategies for malignancy.

**Methods:**

We assessed microRNA-582 (miR-582-5P) expression in colorectal cancer (CRC) specimens and cell lines by real-time PCR. Luciferase reporter assay was used to confirm the target associations. Colony formation assay and anchorage-independent growth assay were used to analyze the effect of miR-582-5P on cell proliferation. Adenomatous polyposis coli (*APC*) gene and protein expression were examined using real-time PCR and western blotting, respectively.

**Results:**

miR-582-5P was upregulated in the CRC specimens and cell lines and targeted the 3′ untranslated region of *APC* directly. miR-582-5P overexpression increased cyclin D1 and c-*MYC* expression, which subsequently induced CRC cell proliferation in an *APC*-dependent manner.

**Conclusions:**

Our findings suggest that miR-582-5P plays an important role in the progression of CRC by inducing proliferation and may identify new targets for anti-cancer treatment.

**Electronic supplementary material:**

The online version of this article (doi:10.1186/s12957-016-0984-4) contains supplementary material, which is available to authorized users.

## Background

Colorectal cancer (CRC) is one of the most prevalent malignant cancers and affects men and women almost equally. Currently, approximately 1.25 million people in China are diagnosed with CRC, and the incidence of CRC increases annually and it will continue to rise in the next few years [1]. Furthermore, CRC is a major cause of cancer-associated mortality, and more than 600,000 patients will die from it every year worldwide [2]. In the USA and Europe, CRC is the second most frequent cancer that leads to death [3]. In recent years, significant advances have been made in targeted therapies of CRC. However, better targeted drugs are required because the effects of existing drugs are not satisfactory.

The adenomatous polyposis coli (*APC*) gene located at 5q21–q22 encodes a tumor suppressor protein that acts as an antagonist of the Wnt/β-catenin pathway, which controls the CRC cell fate during the maintenance phase of tumors in patients [4, 5]. It is also involved in other processes, including cell cycle control and cell migration, adhesion, differentiation, apoptosis, and transcriptional activation [4]. Defects in this gene may cause familial adenomatous polyposis, an autosomal dominant pre-malignant disease that usually progresses to malignancy. *APC* mutation has been found in over 80 % of CRC cases and significantly less frequently in sporadic high microsatellite instability cancers than in low microsatellite instability or microsatellite instability cancers [6]. Furthermore, *APC* mutation-induced upregulation of the survivin/ABK cascade is associated with crypt cell maturation, expansion of proliferative cell populations, and promotion of tumorigenesis [7].

microRNAs (miRNAs) are a class of endogenous, small, non-coding RNAs involved in multiple biological processes. They negatively regulate post-transcriptional gene expression to act as tumor suppressors or oncogenes by binding to the 3′ untranslated region (UTR) of a target gene [8–11]. miRNAs have been widely proposed as potential targets for anti-cancer therapies because a number of findings have indicated that some miRNAs, such as miR-150 [12, 13], miR-153 [13], miR-561 [14], and miR-622 [15], are involved in the development of human CRC. Publicly available algorithms have indicated that miR-582-5P may directly target the 3′ UTR of *APC*. miR-582-5P reduces the proliferation and invasion of human bladder cancer by suppressing the expression of target genes such as protein geranylgeranyltransferase type I beta subunit (*PGGT1B*), leucine-rich repeat kinase 2 (*LRRK2*), and DIX domain containing 1 (*DIXDC1*) [16]. However, the role of miR-582-5P in CRC progression has not been determined. In this study, we searched for the possible relationship between miR-582-5P and *APC* and the role of miR-582-5P in the development of CRC.

## Methods

### Patients and tissues

The eight malignant CRC tissues and matched adjacent noncancerous tissues used in this study were obtained from patients who had undergone surgery at the China-Japan Union Hospital of Jilin University of the People’s Republic of China. The CRC tissues and matched adjacent noncancerous tissues were frozen and stored in liquid nitrogen until used.

### Cell culture

A normal colonic mucosal epithelial cell line (normal control) was isolated and purified from the adjacent noncancerous tissues obtained from the patients. The human CRC cell lines HT29, SW403, SW480, COLO205, SW620, COLO320DM, and KM202L were purchased from American Type Culture Collection (Manassas, VA, USA) and cultured in Dulbecco’s modified Eagle’s medium (Invitrogen, Carlsbad, CA, USA) supplemented with 10 % fetal bovine serum (Invitrogen) at 37 °C in a 5 % CO_2_ atmosphere in a humidified incubator.

### Plasmids and transfection

The human *APC* 3′ UTR was PCR-amplified from genomic DNA from SW480 cells and cloned into pGL3 vectors (Promega, Madison, WI, USA). Transfection of miR-582-5P mimic, miR-582-5P inhibitor (miR-582-5P-in), negative control (NC), NC inhibitor (NC-in) (RiboBio, Guangzhou, China), and plasmids was performed using Lipofectamine 2000 (Invitrogen) according to the manufacturer’s instructions.

### RNA extraction and real-time quantitative PCR

Total miRNA from cultured cells and cancer tissue samples was extracted using the mirVana miRNA Isolation Kit (Ambion, Austin, TX, USA) according to the manufacturer’s manual. The expression level of miR-582-5P was performed using miR-582-5P-specific primer and probe (TaqMan MicroRNA Assay Kit; Applied Biosystems, Foster City, CA, USA) on an ABI 7900 system (Applied Biosystems). The expression of miR-582-5P was defined based on Ct, and relative expression levels were calculated as 2^−[(Ct of miR-582-5p) − (Ct of U6)]^ after normalization with reference to the quantification of *U6* small nuclear RNA expression. The following primers (RiboBio, Guangzhou, China) were synthesized and used in this study: GAPDH forward: 5′-AATCTCCACTTTGCCACTG-3′, GAPDH reverse: 5′-CCTCGTCCCGTAGACAAAA-3′; cyclin D1 forward: 5′-AGGAGAACAAACAGATCA-3′, cyclin D1 reverse: 5′-TAGGACAGGAAGTTGTTG-3′; and c-MYC forward: 5′-TCAAGAGGTGCCACGTCTCC-3′, c-MYC reverse: 5′-TCTTGGCAGCAGGATAGTCCTT-3′.

### Western blotting

Western blotting was performed according to a previously reported method [17]. The membranes were probed with polyclonal mouse antibodies: anti-APC (ab15270; 1:1000; Abcam, Cambridge, UK), anti-cyclin D1 (1:1000; Cell Signaling Technology, Danvers, MA, USA), and anti-c-MYC (1:1000; Cell Signaling Technology). The membranes were stripped and re-probed with anti-α-tubulin mouse monoclonal antibody (1:1000; Cell Signaling Technology) as the loading control.

### Luciferase assay

Cells were seeded in 24-well plates and allowed to settle for 24 h. PGL3-APC-luciferase plasmid or pGL3-Mut-luciferase plasmid (100 ng) was transfected into CRC cells using Lipofectamine 2000 according to the manufacturer’s instructions. Luciferase and control signals were measured 48 h after transfection using a Dual Luciferase Reporter Assay Kit (Promega) according to a protocol provided by the manufacturer. Three independent experiments were performed, and the data are presented as the mean ± SD.

#### 3-(4,5-Dimethyl-2-thiazolyl)-2, 5-diphenyl-2H-tetrazolium bromide assay

Cells were seeded on 96-well plates and stained at the indicated time points with 100 μl sterile 3-(4,5-dimethyl-2-thiazolyl)-2, 5-diphenyl-2H-tetrazolium bromide (MTT) dye (0.5 mg/ml, Invitrogen) for 4 h at 37 °C, followed by the removal of the culture medium and the addition of dimethyl sulfoxide (Sigma-Aldrich, St. Louis, MO, USA). The absorbance at 450 nm was measured using a microplate reader (Bio-Rad, La Jolla, CA, USA). Three independent repeat experiments were performed, and the data are presented as the mean ± SD.

#### Colony formation assay

Cells were seeded on a 6-well plate (1 × 10^3^ cells per well) and cultured for 10 days. The colonies were stained with 1.0 % crystal violet for 5 min after a 15-min fixation with 10 % formaldehyde. All experiments were performed in triplicates.

#### Anchorage-independent growth assay

Five hundred cells were trypsinized and suspended in 2 ml complete medium plus 0.3 % agar (Sigma-Aldrich). The agar-cell mixture was plated on top of a bottom layer containing 1 % complete medium agar mixture. After 10 days, viable colonies that were larger than 0.1 mm (diameter) were counted with an ocular micrometer (Xintu Photonics Co., Ltd, Fuzhou, China). The experiment was performed three times independently for each cell line.

### Statistical analysis

Student’s *t* test was used to evaluate the significant difference between the two groups of data in all pertinent experiments. *P* < 0.05 (Student’s *t* test) was considered statistically significant.

## Results

### miR-582-5P is upregulated in CRC cells and tumor tissues

**Fig. 1:**
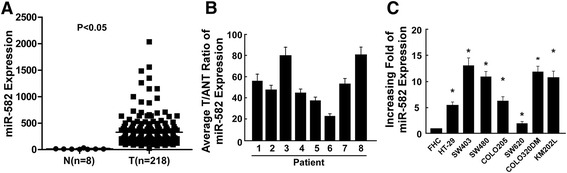
miR-582-5P expression is upregulated in CRC. **a** miR-582-5P expression in 218 CRC tumors and eight matched adjacent noncancerous tissues with CRC tissues based on the TCGA dataset. **b** Real-time PCR analysis of miR-582-5P expression in tumor tissues (*T*) and adjacent noncancerous tissues (*ANT*) from eight patients with CRC. **c** Real-time PCR analysis of miR-582-5P expression in human normal colonic mucosal epithelial cells and CRC cells. Mean miR-582-5P expression was normalized to U6 expression. *Bars* represent the mean ± SD of three independent experiments (*P < 0.05)

To investigate the function of miR-582-5P in the development of human CRC, we analyzed the expression of miR-582-5P in 218 CRC tumors and matched eight adjacent noncancerous tissues with CRC tissues utilizing The Cancer Genome Atlas (TCGA) dataset. As shown in Fig. 1a, miR-582-5P was significantly upregulated in the CRC tumor tissues (*P* < 0.05). Furthermore, miR-582-5P was significantly upregulated in the eight CRC tissue samples as compared with the adjacent noncancerous colorectal tissues (Fig. 1b). Real-time PCR showed that miR-582-5P expression was markedly increased in the seven CRC cell lines as compared with that in the normal colorectal epithelial cells (Fig. 1c). Collectively, our results show that miR-582-5P is overexpressed in the CRC cell lines and tissues.

### miR-582-5P overexpression induces cell proliferation

**Fig. 2:**
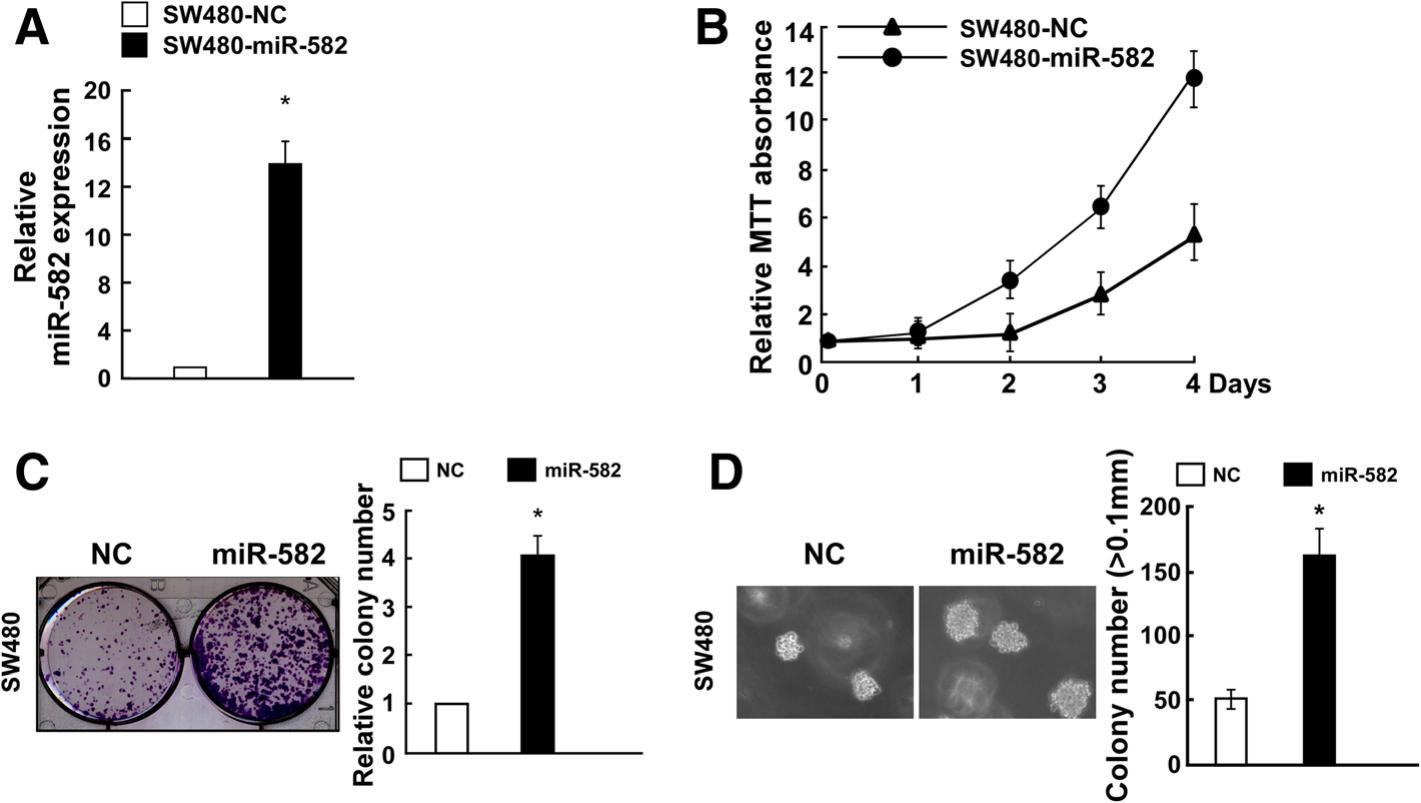
Ectopic miR-582-5P expression induces CRC cell proliferation. **a** Real-time PCR analysis confirming that SW480 cells were successfully transfected with miR-582-5P mimic or NC (negative control) RNA. **b** MTT assay analysis of the effects of ectopic miR-582-5P expression on CRC cell proliferation. **c** Ectopic overexpression of miR-582-5P promoted the colony formation ability of SW480 cells. Representative micrographs (*left*) and quantification (*right*) of crystal violet-stained cell colonies at 10 days after transfection. **d** Anchorage-independent growth assay demonstrating that ectopic overexpression of miR-582-5P promoted SW480 cell tumorigenicity. Shown are representative micrographs (*left*) and quantification of colonies >0.1 mm (*right*). *Bars* represent the mean ± SD of three independent experiments (*P < 0.05)

To investigate whether miR-582-5P plays a role in CRC development and progression, we transfected SW480 cells with miR-582-5P mimic, miR-582-5P-in, NC, and NC-in RNA. As shown in Fig. 2a, the SW480 cells were successfully transfected with miR-582-5P. The MTT assay was used to examine the effect of miR-582-5P overexpression on the CRC cell proliferation and showed that ectopic expression of miR-582-5P increased the cell growth rate significantly (Fig. 2b). Furthermore, miR-582-5P overexpression promoted SW480 cell proliferation in the colony formation assay and anchorage-independent growth assay (Fig. 2c, d). These results suggest that miR-582-5P upregulation increases CRC cell proliferation in vitro.

### Downregulation of miR-582-5P inhibits cell proliferation

**Fig. 3:**
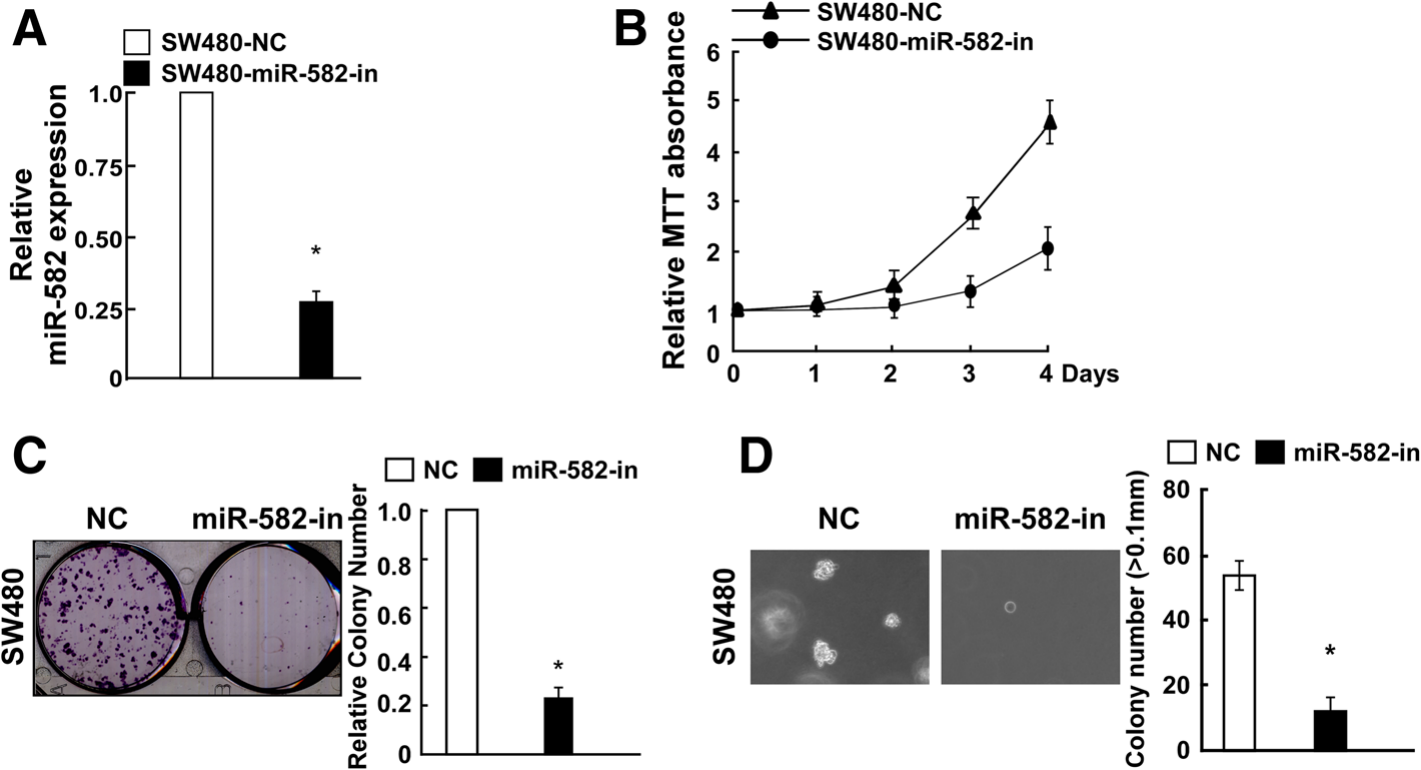
Downregulation of miR-582-5P inhibits CRC cell proliferation. **a** Real-time PCR analysis confirming that SW480 cells were successfully transfected with miR-582-5P-in or NC (negative control) RNA. **b** MTT assay demonstrating that miR-582-5P downregulation reduced SW480 cell proliferation. **c** Downregulation of miR-582-5P reduced the colony formation ability of SW480 cells. Shown are representative micrographs (*left*) and quantification (*right*) of crystal violet-stained cell colonies at 10 days after transfection. **d** Anchorage-independent growth assay demonstrating that miR-582-5P downregulation reduced the tumorigenicity of SW480 cells. Shown are representative micrographs (*left*) and quantification of colonies >0.1 mm (*right*). *Bars* represent the mean ± SD of three independent experiments (*P < 0.05)

Figure 3a shows that SW480 cells were successfully transfected with miR-582-5P-in. The MTT assay demonstrated that inhibiting miR-582-5P significantly reduced SW480 cell growth (Fig. 3b). Furthermore, the downregulation of miR-582-5P inhibited SW480 cell proliferation in the colony formation assay and anchorage-independent growth assay (Fig. 3c, d). Consistent with the findings depicted in Fig. 2, these results suggest that miR-582-5P downregulation reduces CRC cell proliferation in vitro.

### miR-582-5P targets APC in CRC cells directly

**Fig. 4:**
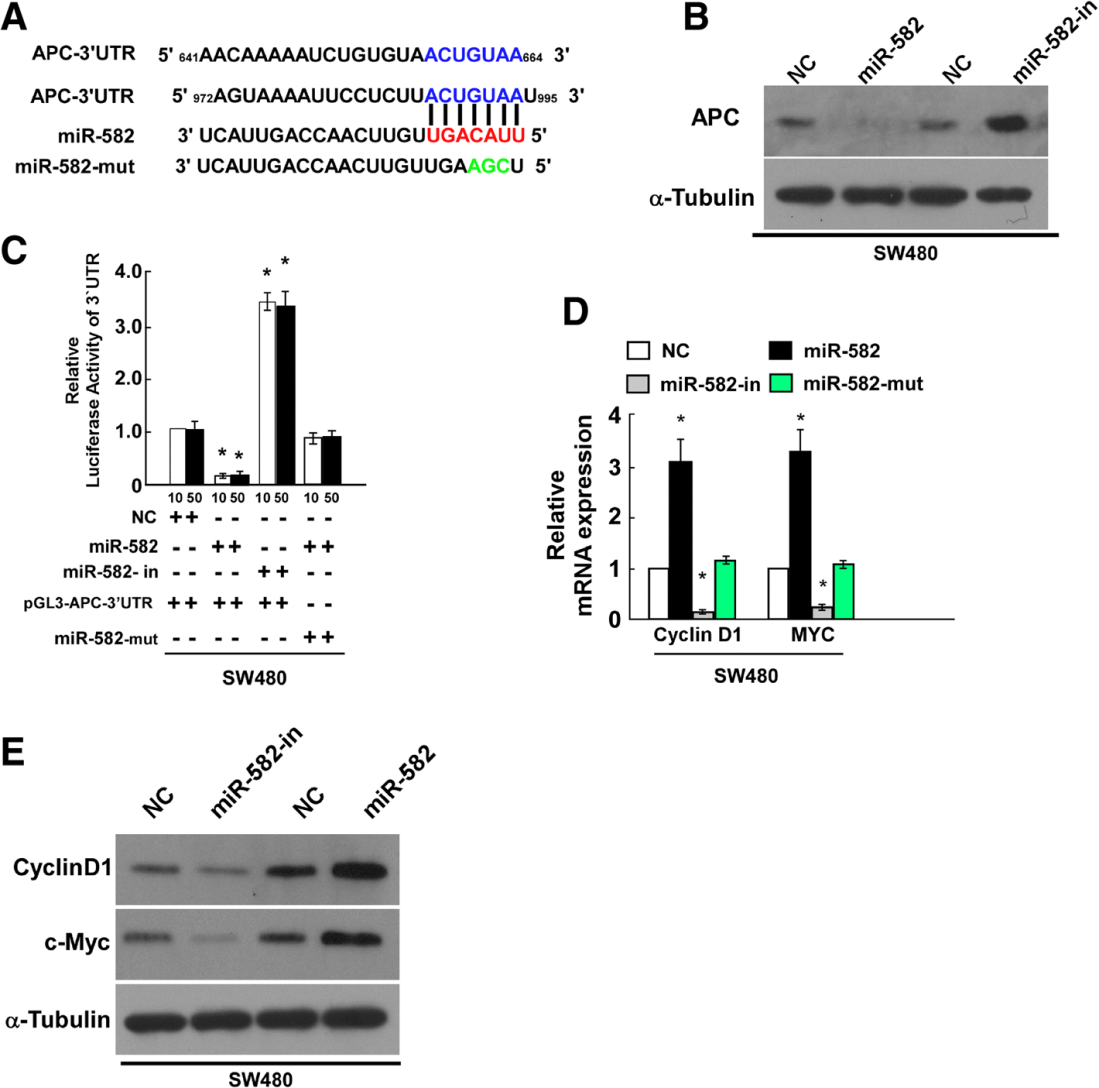
miR-582-5P targets the 3′ UTR of *APC* mRNA directly. **a** Schematic representation of mature miR-582-5P sequence and miR-582-5P target sites of the 3′ UTR of *APC* mRNA (*red*) and the 3′ UTR of *APC* mutant mRNA containing three altered nucleotides (*green*) in the putative target site (APC-3′ UTR-mut). **b** Western blotting analysis of APC (300KD) expression in SW480 cells transfected with miR-582-5P or miR-582-5P-in as compared to NC cells. **c** Luciferase assay of pGL3-APC-3′ UTR reporter co-transfected with 10 or 50 nM miR-582-5P mimic, miR-582-5P-in, or miR-582-5P-mut (pGL3-APC-3′ UTR) in SW480 cells. **d**, **e** Real-time PCR **(d)** and western blotting **(e)** analysis of *cyclin D1* (cyclin D1, 34KD) and c-*MYC* (c-MYC, 53KD) mRNA expression in SW480 cells transfected with miR-582-5P mimic or miR-582-5P-in. *Bars* represent the mean ± SD of three independent experiments (*P < 0.05)

To explore the molecular mechanism of miR-582-5P function in CRC cells, we used publicly available algorithms (TargetScan, PicTar, miRanda) to predict miR-582-5P targets in humans. The results indicated that *APC* was a potential target of miR-582-5P (Fig. 4a). As predicted, western blotting showed that APC expression was decreased in miR-582-5P-overexpressed SW480 cells and was increased in miR-582-5P-downregulated SW480 cells (Fig. 4b). Meanwhile, the results of COLO205 were similar with that of SW480 (Additional file 1: Figure S1).To examine whether miR-582-5P-mediated APC downregulation occurred via the 3′ UTR of *APC*, we subcloned the *APC* 3′ UTR fragment, containing a miR-582-5P binding site, into a pGL3 luciferase reporter vector. miR-582-5P overexpression reduced the luciferase reporter activity of the *APC* 3′ UTR consistently and dose-dependently; miR-582-5P inhibition had the opposite effect. However, *APC* 3′ UTR luciferase reporter activity was unaffected by point mutations in the miR-582-5P-binding seed region (Fig. 4c). Collectively, our results suggest that *APC* is a direct target of miR-582-5P.

Subsequently, real-time PCR demonstrated that miR-582-5P overexpression significantly increased the expression of *cyclin D1* and c-*MYC* mRNA in SW480 cells (Fig. 4d). Additionally, the western blotting results were consistent with the real-time PCR data (Fig. 4e). These findings indicate that miR-582-5P may play an important role in regulating the proliferation of CRC cells.

### miR-582-5P induces SW480 cell proliferation in an APC-dependent manner

**Fig. 5:**
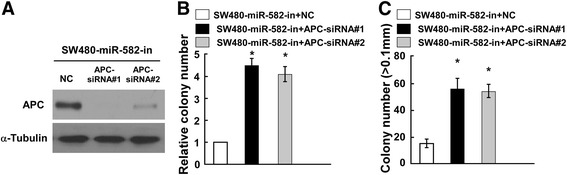
miR-582-5P induces SW480 cell proliferation in an *APC*-dependent manner. **a** Western blotting analysis of APC in miR-582-5P-in SW480 cells transfected with APC siRNA. **b**, **c** Histogram of colony formation assay **(c)** and anchorage-independent growth assay (**b**) on SW480 cells 10 days after inoculation. *Bars* represent the mean ± SD of three independent experiments (*P < 0.05)

We determined the effect of miR-582-5P repression in SW480 cells based on the hypothesis that *APC* repression by miR-582-5P may lead to cell proliferation. We repressed endogenous *APC* expression using an *APC*-specific small interfering RNA (siRNA) (Fig. 5a). The colony formation assay and anchorage-independent growth assay both showed that silencing *APC* in miR-582-5P-in-transfected cells increased cell proliferation (Fig. 5b, c). Together, our results suggest that miR-582-5P may repress *APC* expression and further promote CRC progression. However, the silenced APC expression, and its effect on cell proliferation, is reversed in miR-582-5P-in-transfected cells. These data show that miR-582-5P induces SW480 cell proliferation by repressing *APC* expression and that *APC* may play an important role in miR-582-5P-mediated cell proliferation.

## Discussion

microRNAs, a class of small regulatory RNA molecules that negatively regulate their mRNA targets in a sequence-specific manner, play important roles in multiple biological processes such as cell differentiation, proliferation, oncogenesis, angiogenesis, invasion, and metastasis and may function as either tumor suppressors or oncogenes [18–20]. Uchino et al. found that miR-582-5P reduced human bladder cancer proliferation and invasion by suppressing the expression of target genes such as *PGGT1B*, *LRRK2*, and *DIXDC1* [16]. Furthermore, Floyd et al. found that miR-582-5P directly targeted caspase 3 (*CASP3*), caspase 9 (*CASP9*), and *BIM*, consequently influencing glioblastoma cell survival [21]. Liu et al. suggested that miR-582-5P can inhibit monocyte apoptosis by downregulating forkhead box O1 (*FOXO1*) expression and that it plays an important role in regulating anti-*Mycobacterium tuberculosis*-directed immune responses [22]. However, there has been no research on the role of miR-582-5P in CRC progression. In our study, we found that miR-582-5P was significantly overexpressed in CRC cells as compared to normal colonic mucosa epithelial cells. Additionally, miR-582-5P was upregulated in CRC tissues when compared with the matched adjacent noncancerous tissues. Furthermore, ectopic expression of miR-582-5P significantly increased cell growth, while inhibiting miR-582-5P had the opposite effect. These results indicate that upregulation of miR-582-5P may correlate with the progression of CRC and that miR-582-5P may function as an onco-miRNA in CRC.

Mutations in *APC* are believed to be one of the earliest events that contribute to CRC initiation. *APC* I1307K germline mutation is the most common and important mutation. Firstly, the *APC* I1307K germline mutation is associated with increased risk for CRC development, tumor location, and tumor stage [23]. Secondly, compared with non-carriers, *APC* I1307K carriers had increased numbers of adenomas and tumors per patient, as well as younger age at diagnosis. The *APC* I1307K allele is associated with an estimated relative risk of 1.5–1.7 for colorectal neoplasia [24]. However, Figer et al. suggested that these mutations contribute little to disease pathogenesis [25, 26]. In addition, methylation of the *APC* gene promoter region in cancerous tissue in combination with the predominance of methylation in normal tissue may serve as a prognostic factor in patients with CRC [27, 28]. In this study, we used three methods to confirm that *APC* is a predicted target gene of miR-582-5P. Western blotting showed that overexpression of miR-582-5P downregulated APC protein expression but upregulated cyclin D1 and c-MYC expression. The luciferase activity assay and point mutation analysis demonstrated that *APC* downregulation was mediated by miR-582-5P specifically targeting the *APC* 3′ UTR. The mechanism of miR-582-5P induction of cell proliferation is currently under investigation in our laboratory.

## Conclusions

Our results show that miR-582-5P is markedly upregulated in the CRC cells and clinical tissues as compared with matched adjacent noncancerous tissues from the same patient. Furthermore, *APC* is a direct target gene of miR-582-5P, and overexpression of miR-582-5P reduced the expression of *APC* and inhibited CRC cell proliferation, whereas downregulation had the opposite effect. Further investigation is required to fully characterize the biological function of miR-582-5P and its clinical relevance in the development of CRC. Collectively, although the precise mechanisms are not yet fully understood, this finding suggests that miR-582-5P may play an important role in regulating the proliferation of CRC cells and represent a therapeutic target for colorectal cancer.

## Additional file

Additional file 1: Figure S1. Real-time PCR (left) and western blotting analysis (right) of APC expression in COLO205 cells transfected with miR-582-5P or miR-582-5P-in as compared to NC cells. (TIF 109 kb)
